# Supplementary material for: Agreement and differences between the equations for estimating muscle and bone mass using the anthropometric method in recreational strength trainees
Source: PeerJ. 2024 Jun 7;12:e17506. doi: 10.7717/peerj.17506 (PMC11164060; doi:10.7717/peerj.17506)
Supplement: Supplemental Information 3 [file peerj-12-17506-s003.docx]

**Questionnaire project "Agreement and differences between the equations for estimating muscle and bone mass using the anthropometric method in recreational strength trainees".**

Dear,

We inform you that from the International Chair of Kinanthropometry and the Research Group on Prevention of Injuries in Sport (PRELEDE) of the Catholic University of Murcia, we are going to develop a project entitled " Agreement and differences between the equations for estimating muscle and bone mass using the anthropometric method in recreational strength trainees". In it we intend to observe the muscle mass and bone mass obtained by different equations derived from kinanthropometry, and to know the different factors that may influence their comparability.

To begin your participation as a volunteer in this project, please fill in the questionnaire below. Please answer honestly as the data will be treated in a completely anonymous way. Thank you for your participation.

NOTE: Completion of this questionnaire implies that I have been informed of the study and research procedures of the Project entitled: " Agreement and differences between the equations for estimating muscle and bone mass using the anthropometric method in recreational strength trainees" and of the research procedures. Completion of this questionnaire will be considered informed consent to participate in this research. The data collected will be treated in compliance with Regulation (EU) 2016/679 of the European Parliament and of the Council of 27 April 2016, Royal Decree-Law 5/2018 of 27 July and Organic Law 15/1999 of 13 December on the Protection of Personal Data.

**PERSONAL INFORMATION**

1. Participant's code: _______________________________________________________
2. Current Date: ___________________________________________________________
3. Date of Birth: ___________________________________________________________
4. Sex:
5. Male (go to question 6)
6. Female (go to question 5)
7. Otro (go to question 6)
8. In case you answered "female", indicate the date when your last menstruation started: _______________________________________________________________________

**MEDICAL HISTORY**

1. Do you suffer or have you suffered in the last 6 months from any illness?
2. Yes (go to question 7)
3. No (go to question 8)
4. Indicate which illness(es) and the date of the illness: _______________________________________________________________________
5. Have you suffered any type of injury in the last 6 months?
6. Yes (go to question 9)
7. No (go to question 10)
8. Indicate what type of injury and date of injury:

_______________________________________________________________________

1. Do you take any medication regularly?
2. Yes (go to question 11)
3. No (go to question 12)
4. Indicate which medicine/s and since when:

_______________________________________________________________________

1. Are you taking any hormonal treatment?
2. Yes (go to question 13)
3. No (go to question 14)
4. Indicate what type of treatment and since when:

_______________________________________________________________________

1. Have you had any type of surgery?
2. Yes (go to question 15)
3. No (go to question 16)
4. Which surgery and when was it performed?:

_______________________________________________________________________

**SPORTS HISTORY**

1. Do you practice any type of physical exercise/sport on a regular basis?
2. Yes (go to question 17)
3. No (go to question 21)
4. What type of physical exercise/sport? If several, please indicate them:

_______________________________________________________________________

1. How many years have you been practicing physical exercise/sports on a regular basis?

_______________________________________________________________________

1. How many days a week do you practice physical exercise/sports? Which days?

_______________________________________________________________________

1. What is the average duration of training/session per day (hours/day)?

_______________________________________________________________________

1. **FOOD**

*24-hour dietary recall*

The purpose of this section is to know your food and drink consumption in the last 24 hours.

Write down as accurately as possible all the food and beverages (including water) consumed during yesterday. You can start with the first intake of the day and continue forward in time to complete the whole day. Also note the foods and beverages consumed between meals. You should complete a chart for each hour that you ate or ingested something. When you have finished for the day, leave the remaining boxes blank.

Describe in as much detail as possible the food/drink you consumed (name of food/drink, type and brand; example: cow's milk, skim milk, Hacendado brand). Estimate the amount consumed in household measures or in servings (large, medium, small; example: 1 large cup of milk; 1 deep dish to the top; 2 cases). Indicate whether the amount refers to the raw or cooked food; the whole food (as purchased in the market) or the edible part (e.g., peeled orange). Record the method of preparation used (cooked, fried, roasted, etc.).

Do not forget to note the oil used in the preparations (quantity and type, example: a large spoonful of extra virgin olive oil), bread, sugar or beverages consumed (water, soft drinks, milk, coffees, alcoholic beverages, etc.).

*Complete example:*

Meal/Drink 1:

- - Time: 07:00 am.
  - Place of intake: Kitchen in my home.
  - Food or drink ingested: Iberian ham, tomato and cheese toast; and a glass of cold milk with sugar.
  - Brands of products: Toasted sliced bread (Bimbo), Iberian ham (Hacendado), light cheese slices (Hacendado), tomato (mercado), skim milk (Hacendado), sugar (hacendado).
  - Description of preparation method: All uncooked.
  - Quantity consumed. 2 medium slices of bread, 2 slices of Iberian ham, 1 medium slice of light cheese, half a large tomato, 1 large cup of skimmed milk, 1 small spoonful of sugar.

Meal/Drink 2:

- - Time: 10:00 am.
  - Place of intake: University.
  - Food or drink ingested: Water.
  - Brands of products: Lanjarón.
  - Description of method of preparation: Not applicable.
  - Amount consumed. 2 glasses of water (half a liter).

1. From here, complete your information:

Food/Drink 1:

- - Time.
  - Place of intake.
  - Food or drink ingested.
  - Brands of the products.
  - Description of method of preparation.
  - Quantity consumed.

Food/Drink 2:

- - Time.
  - Place of intake.
  - Food or drink ingested.
  - Brands of the products.
  - Description of method of preparation.
  - Quantity consumed.

Food/Drink 3:

- - Time.
  - Place of intake.
  - Food or drink ingested.
  - Brands of the products.
  - Description of method of preparation.
  - Quantity consumed.

Food/Drink 4:

- - Time.
  - Place of intake.
  - Food or drink ingested.
  - Brands of the products.
  - Description of method of preparation.
  - Quantity consumed.

Food/Drink 5:

- - Time.
  - Place of intake.
  - Food or drink ingested.
  - Brands of the products.
  - Description of method of preparation.
  - Quantity consumed.

Food/Drink 6:

- - Time.
  - Place of intake.
  - Food or drink ingested.
  - Brands of the products.
  - Description of method of preparation.
  - Quantity consumed.

Food/Drink 7:

- - Time.
  - Place of intake.
  - Food or drink ingested.
  - Brands of the products.
  - Description of method of preparation.
  - Quantity consumed.

Food/Drink 8:

- - Time.
  - Place of intake.
  - Food or drink ingested.
  - Brands of the products.
  - Description of method of preparation.
  - Quantity consumed.

Food/Drink 9:

- - Time.
  - Place of intake.
  - Food or drink ingested.
  - Brands of the products.
  - Description of method of preparation.
  - Quantity consumed.

Food/Drink 10:

- - Time.
  - Place of intake.
  - Food or drink ingested.
  - Brands of the products.
  - Description of method of preparation.
  - Quantity consumed.

Food/Drink 11:

- - Time.
  - Place of intake.
  - Food or drink ingested.
  - Brands of the products.
  - Description of method of preparation.
  - Quantity consumed.

Food/Drink 12:

- - Time.
  - Place of intake.
  - Food or drink ingested.
  - Brands of the products.
  - Description of method of preparation.
  - Quantity consumed.

1. **CONSUMPTION OF SPORTS SUPPLEMENTS**

The following questionnaire is about your consumption of sports supplements.

Indicate the name of the supplement consumed (example: Creatine), brand of the supplement (example: Proxis), format consumed (powder, tablets, etc.). Mention the frequency of consumption (daily, weekly, monthly), dosage (1 gram, 10,000 IU, 1 tablet), time of consumption (on an empty stomach before breakfast, before training, etc.), time since consuming the supplement (1 year ago, 1 month, etc.) and reason for consuming the supplement (muscle mass gain, fat loss, etc.).

These are some of the products considered sports supplements:

- - Multivitamins and minerals: vitamin C, D, B12, multivitamin complexes.
  - Creatine.
  - Caffeine: Mention only its consumption in tablets or different format to the drink.
  - Protein powder.
  - Pre-workout.
  - Sports drinks.
  - Energy bars.
  - Diuretics.
  - Beta alanine.
  - Glycerol.

* If you consider that you consume a product that is not among those mentioned, please include it describing the requested aspects.

Below is an example of how you should complete this section:

- - Supplement name: Creatine.
  - Brand: Proxis.
  - Format consumed: Powder.
  - Frequency of consumption: Daily.
  - Dosage: 5 gr/day
  - Moment of consumption: Pre-workout.
  - Since consumed: 4 months ago.

Reason for use: To increase muscle mass.

1. Do you consume any sports supplement? *
2. Yes (go to question 25).
3. No (go to question 26).
4. From here, complete your information. Fill in one box for each supplement. Leave blank any boxes you do not need:

Supplement 1.

- Name of supplement.
- Brand.
- Format consumed.
- Frequency of consumption.
- Dose.
- Time of consumption.
- Time since it has been consumed.
- Reason for consumption.

Supplement 2.

- Name of supplement.
- Brand.
- Format consumed.
- Frequency of consumption.
- Dose.
- Time of consumption.
- Time since it has been consumed.
- Reason for consumption.

Supplement 3.

- Name of supplement.
- Brand.
- Format consumed.
- Frequency of consumption.
- Dose.
- Time of consumption.
- Time since it has been consumed.
- Reason for consumption.

Supplement 4.

- Name of supplement.
- Brand.
- Format consumed.
- Frequency of consumption.
- Dose.
- Time of consumption.
- Time since it has been consumed.
- Reason for consumption.

Supplement 5.

- Name of supplement.
- Brand.
- Format consumed.
- Frequency of consumption.
- Dose.
- Time of consumption.
- Time since it has been consumed.
- Reason for consumption.

Supplement 6.

- Name of supplement.
- Brand.
- Format consumed.
- Frequency of consumption.
- Dose.
- Time of consumption.
- Time since it has been consumed.
- Reason for consumption.

Supplement 7.

- Name of supplement.
- Brand.
- Format consumed.
- Frequency of consumption.
- Dose.
- Time of consumption.
- Time since it has been consumed.
- Reason for consumption.

Supplement 8.

- Name of supplement.
- Brand.
- Format consumed.
- Frequency of consumption.
- Dose.
- Time of consumption.
- Time since it has been consumed.
- Reason for consumption.

Supplement 9.

- Name of supplement.
- Brand.
- Format consumed.
- Frequency of consumption.
- Dose.
- Time of consumption.
- Time since it has been consumed.
- Reason for consumption.

Supplement 10.

- Name of supplement.
- Brand.
- Format consumed.
- Frequency of consumption.
- Dose.
- Time of consumption.
- Time since it has been consumed.
- Reason for consumption.

**QUESTIONNAIRE OF PHYSICAL EXERCISE IN THE PREVIOUS 24 HOURS.**

1. Did you train yesterday?
2. Yes (go to question 27).
3. No (you have finished the questionnaire).
4. What activity did you do?

_______________________________________________________________________

1. According to the scale below, what value of perceived exertion would you give to the intensity of your activity?

_______________________________________________________________________


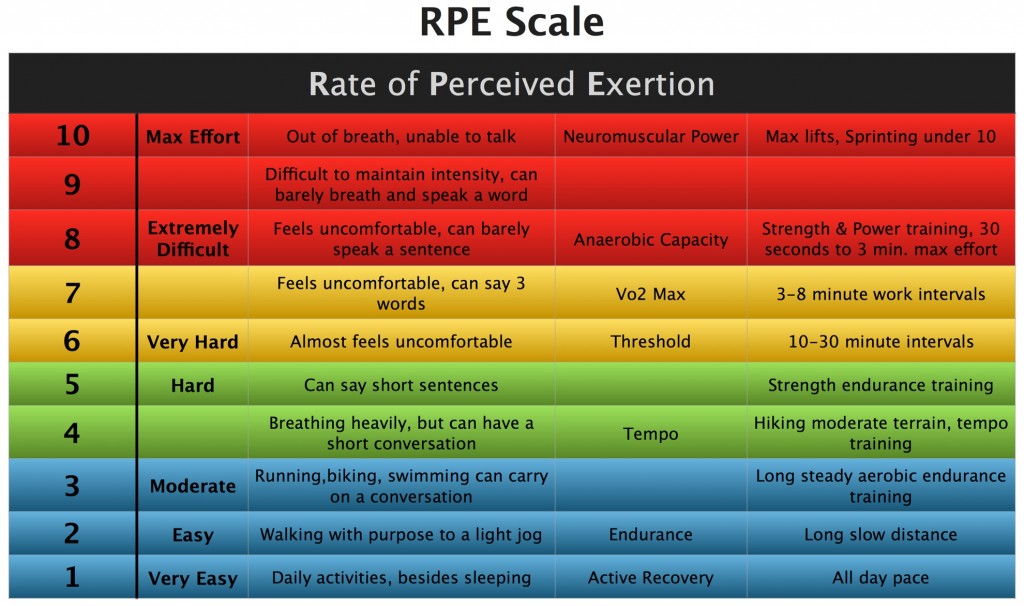


1. How long did you train for (in minutes)?

_______________________________________________________________________
