# Supplementary material for: Agreement and differences between the equations for estimating muscle and bone mass using the anthropometric method in recreational strength trainees
Source: PeerJ. 2024 Jun 7;12:e17506. doi: 10.7717/peerj.17506 (PMC11164060; doi:10.7717/peerj.17506)
Supplement: Supplemental Information 4 [file peerj-12-17506-s004.docx]

**Cuestionario proyecto “Diferencias entre las ecuaciones de estimación de la masa muscular y ósea mediante el método antropométrico en practicantes de fuerza recreativos”**

Estimado/a voluntario/a,

Le informamos que desde la Cátedra Internacional de Cineantropometría y del Grupo de Investigación de Prevención de Lesiones en el Deporte (PRELEDE) de la Universidad Católica de Murcia, se va a desarrollar un proyecto titulado "Diferencias entre las ecuaciones de estimación de la masa muscular y ósea mediante el método antropométrico en practicantes de fuerza recreativos". En él pretendemos observar la masa muscular y masa ósea obtenida por diferentes ecuaciones derivadas de la cineantropometría, y conocer los distintos factores que pueden influir en la comparabilidad de las mismas.

Para comenzar su participación como voluntario/a en dicho proyecto, debe cumplimentar el cuestionario que encontrará a continuación. Por favor, responda con sinceridad ya que los datos serán tratados de manera totalmente anónima. Gracias por su participación.

NOTA: La cumplimentación de este cuestionario implica que he sido informado/a del estudio y procedimientos de la investigación del Proyecto titulado: "Diferencias entre las ecuaciones de estimación de la masa muscular y ósea mediante el método antropométrico en practicantes de fuerza recreativos" y de los procedimientos de la investigación. La cumplimentación del presente cuestionario será considerada el consentimiento informado para participar en la presente investigación. Los datos recogidos serán tratados en cumplimiento con el Reglamento (UE) 2016/679 del Parlamento Europeo y del Consejo, de 27 de abril de 2016, el Real Decreto-Ley 5/2018, de 27 de julio y Ley Orgánica 15/1999, de 13 de diciembre, de Protección de Datos de Carácter Personal.

**DATOS PERSONALES**

1. Código del participante: ___________________________________________________
2. Fecha actual: ___________________________________________________________
3. Fecha de Nacimiento: ____________________________________________________
4. Sexo:
5. Masculino (Pase a la pregunta 6)
6. Femenino (conteste la pregunta 5)
7. Otro (Pase a la pregunta 6)
8. En caso de haber respondido “femenino”, indique la fecha de inicio de su última menstruación: __________________________________________________________

**HISTORIAL MÉDICO**

1. ¿Sufre o ha sufrido en los últimos 6 meses alguna enfermedad?
2. Sí (conteste la pregunta 7).
3. No (Pase a la pregunta 8).
4. Indique qué enfermedad/es y fecha de la misma: ______________________________
5. ¿Ha sufrido algún tipo de lesión en los últimos 6 meses?
6. Sí (conteste la pregunta 9).
7. No (pase a la pregunta 10).
8. Indique qué tipo de lesión y fecha de la misma: ________________________________
9. ¿Toma algún medicamento con regularidad?
10. Sí (conteste la pregunta 11).
11. No (Pase a la pregunta 12).
12. Indiqué qué medicamento/s y desde cuándo: _________________________________
13. ¿Está tomando algún tratamiento hormonal?
14. Sí (conteste la pregunta 13).
15. No (pase a la pregunta 14).
16. Indique qué tipo de tratamiento y desde cuándo: ______________________________
17. ¿Se le ha realizado algún tipo de cirugía?
18. Sí (pase a la pregunta 15).
19. No (Pase a la pregunta 16).
20. ¿Qué cirugía? ¿Cuándo fue? : _______________________________________________

**HISTORIAL DEPORTIVO**

1. ¿Práctica algún tipo de ejercicio físico/deporte de forma regular?
2. Sí (continúe con la pregunta 17).
3. No (pase a la pregunta 18).
4. ¿Qué tipo de ejercicio físico/deporte? Si son varios, indíquelos: ____________________
5. ¿Hace cuantos años practica ejercicio físico/deporte de manera regular?: ____________
6. ¿Cuántos días a la semana realiza ejercicio físico/deporte? ¿Qué días?: ______________
7. ¿Cuál es la duración promedio del entrenamiento/sesión por día? (horas/día): ________

**ALIMENTACIÓN**

*Cuestionario de alimentación 24hs*

El objetivo de esta sección es conocer tu consumo de alimentos y bebidas en las últimas 24 horas.

Anota con la mayor precisión posible todos los alimentos y bebidas (incluido el agua) consumidos durante el día de ayer. Puedes empezar con la primera ingesta del día y continuar hacía delante en el tiempo hasta completar el día entero. Anota también los alimentos y bebidas consumidos entre horas. Debes completar un cuadro por cada hora la que comiste o ingeriste algo. Cuando hayas terminado el día, deja en blanco los cuadros restantes.

Describa lo más detalladamente posible el alimento/bebida que consumiste (nombre del alimento/bebida, tipo y marca; ejemplo: leche de vaca, desnatada, marca Hacendado). Estima la cantidad consumida en medidas caseras o en raciones (grande, mediana, pequeña, ejemplo: 1 taza de leche grande; 1 plato hondo hasta arriba; 2 casos). Indique si la cantidad se refiere al alimento crudo o cocinado; al alimento entero (tal como se compra en el mercado) o a la parte comestible (ej. naranja pelada). Registra el método de preparación empleado (cocido, frito, asado, etc.).

No olvides anotar el aceite empleado en las preparaciones (cantidad y tipo, ejemplo: una cucharada grande de aceite de oliva extra virgen), el pan, el azúcar o las bebidas consumidas (agua, refrescos, leche, cafés, bebidas alcohólicas, etc.).

*Ejemplo completo:*

Comida/Bebida 1:

- - Hora: 07:00 am.
  - Lugar de la ingesta: Cocina de mi casa.
  - Comida o bebida ingerida: Tostadas de jamón ibérico, tomate y queso; y un vaso de leche fría con azúcar.
  - Marcas de los productos: Pan de molde tostado (Bimbo), jamón ibérico (Hacendado), fetas de queso light (Hacendado), tomate (mercado), leche desnatada (Hacendado), azúcar (hacendado).
  - Descripción del método de preparación: Todo sin cocinar.
  - Cantidad consumida. 2 rebanadas medianas de pan, 2 lonchas de jamón ibérico, 1 feta mediana de queso light, medio tomate grande, 1 taza grande de leche desnatada, 1 cucharada pequeña de azúcar.

Comida/Bebida 2:

- - Hora: 10:00 am.
  - Lugar de la ingesta: Universidad.
  - Comida o bebida ingerida: Agua.
  - Marcas de los productos: Lanjarón.
  - Descripción del método de preparación: No procede.
  - Cantidad consumida. 2 vasos de agua (medio litro).

1. A partir de aquí, completa tu información:

Comida/Bebida 1:

- - Hora.
  - Lugar de la ingesta.
  - Comida o bebida ingerida.
  - Marcas de los productos.
  - Descripción del método de preparación.
  - Cantidad consumida.

Comida/Bebida 2:

- - Hora.
  - Lugar de la ingesta.
  - Comida o bebida ingerida.
  - Marcas de los productos.
  - Descripción del método de preparación.
  - Cantidad consumida.

Comida/Bebida 3:

- - Hora.
  - Lugar de la ingesta.
  - Comida o bebida ingerida.
  - Marcas de los productos.
  - Descripción del método de preparación.
  - Cantidad consumida.

Comida/Bebida 4:

- - Hora.
  - Lugar de la ingesta.
  - Comida o bebida ingerida.
  - Marcas de los productos.
  - Descripción del método de preparación.
  - Cantidad consumida.

Comida/Bebida 5:

- - Hora.
  - Lugar de la ingesta.
  - Comida o bebida ingerida.
  - Marcas de los productos.
  - Descripción del método de preparación.
  - Cantidad consumida.

Comida/Bebida 6:

- - Hora.
  - Lugar de la ingesta.
  - Comida o bebida ingerida.
  - Marcas de los productos.
  - Descripción del método de preparación.
  - Cantidad consumida.

Comida/Bebida 7:

- - Hora.
  - Lugar de la ingesta.
  - Comida o bebida ingerida.
  - Marcas de los productos.
  - Descripción del método de preparación.
  - Cantidad consumida.

Comida/Bebida 8:

- - Hora.
  - Lugar de la ingesta.
  - Comida o bebida ingerida.
  - Marcas de los productos.
  - Descripción del método de preparación.
  - Cantidad consumida.

Comida/Bebida 9:

- - Hora.
  - Lugar de la ingesta.
  - Comida o bebida ingerida.
  - Marcas de los productos.
  - Descripción del método de preparación.
  - Cantidad consumida.

Comida/Bebida 10:

- - Hora.
  - Lugar de la ingesta.
  - Comida o bebida ingerida.
  - Marcas de los productos.
  - Descripción del método de preparación.
  - Cantidad consumida.

Comida/Bebida 11:

- - Hora.
  - Lugar de la ingesta.
  - Comida o bebida ingerida.
  - Marcas de los productos.
  - Descripción del método de preparación.
  - Cantidad consumida.

Comida/Bebida 12:

- - Hora.
  - Lugar de la ingesta.
  - Comida o bebida ingerida.
  - Marcas de los productos.
  - Descripción del método de preparación.
  - Cantidad consumida.

**CONSUMO DE SUPLEMENTOS DEPORTIVOS**

El siguiente cuestionario trata sobre su consumo de suplementos deportivos.

Indique el nombre del suplemento consumido (ejemplo: Creatina), marca del suplemento (ejemplo: Proxis), formato consumido (en polvo, comprimidos, etc.). Mencione la frecuencia de su consumo (diaria, semanal, mensual), dosis (1 gramos, 10.000 UI, 1 comprimido), momento de su consumo (en ayunas previo a desayunar, antes del entrenamiento, etc.), tiempo desde que consume el suplemento (hace 1 año, 1 mes, etc.) y razón por la que consume el suplemento (aumento de masa muscular, descenso de grasa, etc.).

Estos son algunos de los productos considerados suplementos deportivos:

- - Multivitamínicos y minerales: Vitamina C, D, B12, complejos multivitamínicos.
  - Creatina.
  - Cafeína: Mencionar sólo su consumo en comprimidos o formato diferente a la bebida.
  - Proteína en polvo.
  - Pre entrenos.
  - Bebidas deportivas: Gatorade, Powerade.
  - Barras energéticas.
  - Diuréticos.
  - Beta alanina.
  - Glicerol.

* Si considera que consume un producto que no se encuentra dentro de los mencionados inclúyalo describiendo los aspectos solicitados.

A continuación se presenta un ejemplo de cómo tiene que completar esta sección:

- - Nombre del suplemento: Creatina.
  - Marca: Proxis.
  - Formato consumido: En polvo.
  - Frecuencia de consumo: Diario.
  - Dosis: 5 gr/día
  - Momento de consumo: Pre entreno.
  - Tiempo desde que hace que lo consume: hace 4 meses.
  - Razón de su consumo: Aumentar masa muscular.

1. ¿Consume algún suplemento deportivo? *
2. Sí (pase a la pregunta 23).
3. No (pase a la pregunta 24).
4. : A partir de aquí, completa tu información. Rellene un cuadro por cada suplemento. Deje en blanco los cuadros que no necesite:

Suplemento 1.

- -Nombre del suplemento.
- -Marca.
- -Formato consumido.
- -Frecuencia de consumo.
- -Dosis.
- -Momento de consumo.
- -Tiempo desde que hace que lo consume.
- Razón de su consumo.

Suplemento 2.

- -Nombre del suplemento.
- -Marca.
- -Formato consumido.
- -Frecuencia de consumo.
- -Dosis.
- -Momento de consumo.
- -Tiempo desde que hace que lo consume.
- Razón de su consumo.

Suplemento 3.

- -Nombre del suplemento.
- -Marca.
- -Formato consumido.
- -Frecuencia de consumo.
- -Dosis.
- -Momento de consumo.
- -Tiempo desde que hace que lo consume.
- Razón de su consumo.

Suplemento 4.

- -Nombre del suplemento.
- -Marca.
- -Formato consumido.
- -Frecuencia de consumo.
- -Dosis.
- -Momento de consumo.
- -Tiempo desde que hace que lo consume.
- Razón de su consumo.

Suplemento 5.

- -Nombre del suplemento.
- -Marca.
- -Formato consumido.
- -Frecuencia de consumo.
- -Dosis.
- -Momento de consumo.
- -Tiempo desde que hace que lo consume.
- Razón de su consumo.

Suplemento 6.

- -Nombre del suplemento.
- -Marca.
- -Formato consumido.
- -Frecuencia de consumo.
- -Dosis.
- -Momento de consumo.
- -Tiempo desde que hace que lo consume.
- Razón de su consumo.

Suplemento 7.

- -Nombre del suplemento.
- -Marca.
- -Formato consumido.
- -Frecuencia de consumo.
- -Dosis.
- -Momento de consumo.
- -Tiempo desde que hace que lo consume.
- Razón de su consumo.

Suplemento 8.

- -Nombre del suplemento.
- -Marca.
- -Formato consumido.
- -Frecuencia de consumo.
- -Dosis.
- -Momento de consumo.
- -Tiempo desde que hace que lo consume.
- Razón de su consumo.

Suplemento 9.

- -Nombre del suplemento.
- -Marca.
- -Formato consumido.
- -Frecuencia de consumo.
- -Dosis.
- -Momento de consumo.
- -Tiempo desde que hace que lo consume.
- Razón de su consumo.

Suplemento 10.

- -Nombre del suplemento.
- -Marca.
- -Formato consumido.
- -Frecuencia de consumo.
- -Dosis.
- -Momento de consumo.
- -Tiempo desde que hace que lo consume.
- Razón de su consumo.

**CUESTIONARIO DE EJERCICIO FÍSICO DE LAS 24 HORAS PREVIAS.**

1. ¿Entrenaste el día de ayer?
2. Sí (pase a la pregunta 25).
3. No (ha finalizado el cuestionario).
4. ¿Qué actividad realizaste?: _________________________________________________
5. Según la escala de abajo, ¿Qué valor de percepción del esfuerzo le darías a la intensidad de tu actividad?: _________________________________________________________


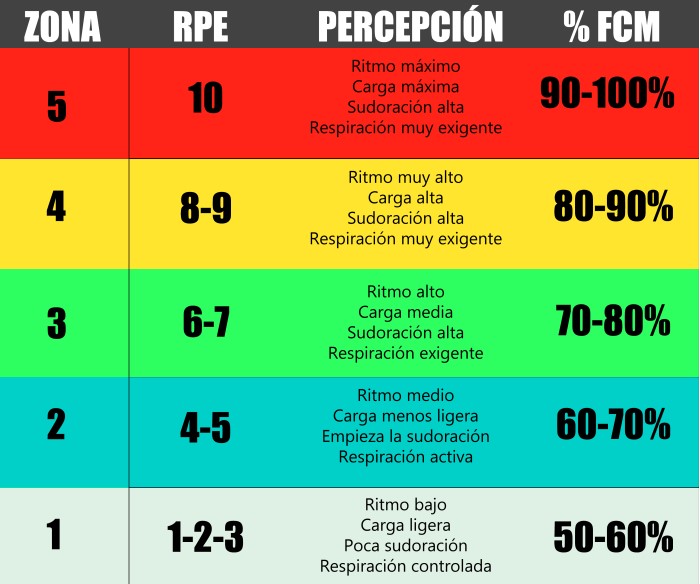


1. ¿Cuánto tiempo entrenaste? (en minutos): ____________________________________
